# Numerical model for static chamber measurement of multi-component landfill gas emissions and its application

**DOI:** 10.1007/s11356-022-20951-2

**Published:** 2022-05-30

**Authors:** Haijian Xie, Xinru Zuo, Yunmin Chen, Huaxiang Yan, Junjun Ni

**Affiliations:** 1 MOE Key Laboratory of Soft Soils and Geoenvironmental Engineering, Zhejiang University, Hangzhou, 310058 China; 2 Center for Balance Architecture, Zhejiang University, 148 Tianmushan Road, Hangzhou, 310007 China; 3 Department of Mechanical, Aerospace and Civil Engineering, School of Engineering, The University of Manchester, Manchester, M13 9PL UK; 4 Department of Civil and Environmental Engineering, The Hong Kong University of Science and Technology, Hong Kong, People’s Republic of China

**Keywords:** Landfill gas, Numerical model, Landfill cover, Static chamber, Relative error, Multi-components

## Abstract

The quantitative assessment of landfill gas emissions is essential to assess the performance of the landfill cover and gas collection system. The relative error of the measured surface emission of landfill gas may be induced by the static flux chamber technique. This study aims to quantify effects of the size of the chamber, the insertion depth, pressure differential on the relative errors by using an integrated approach of in situ tests, and numerical modeling. A field experiment study of landfill gas emission is conducted by using a static chamber at one landfill site in Xi’an, Northwest China. Additionally, a two-dimensional axisymmetric numerical model for multi-component gas transport in the soil and the static chamber is developed based on the dusty-gas model (DGM). The proposed model is validated by the field data obtained in this study and a set of experimental data in the literature. The results show that DGM model has a better capacity to predict gas transport under a wider range of permeability compared to Blanc’s method. This is due to the fact that DGM model can explain the interaction among gases (e.g., CH_4_, CO_2_, O_2_, and N_2_) and the Knudsen diffusion process while these mechanisms are not included in Blanc’s model. Increasing the size and the insertion depth of static chambers can reduce the relative error for the flux of CH_4_ and CO_2_. For example, increasing the height of chambers from 0.55 to 1.1 m can decrease relative errors of CH_4_ and CO_2_ flux by 17% and 18%, respectively. Moreover, we find that gas emission fluxes for the case with positive pressure differential (*∆P*_*in-out*_) are greater than that of the case without considering pressure fluctuations. The Monte Carlo method was adopted to carry out the statistical analysis for quantifying the range of relative errors. The agreement of the measured field data and predicted results demonstrated that the proposed model has the capacity to quantify the emission of landfill gas from the landfill cover systems.

## Introduction

Methane (CH_4_) and carbon dioxide (CO_2_) are the main components of landfill gas (LGF) produced from Municipal Solid Waste (MSW) landfills (Xu et al. 2003; Feng et al. 2019). The quantitative assessment of landfill gas emissions is essential to assess the performance of landfill cover and gas collection systems (Feng et al., 2017; Bian et al. 2021; Fallah and Torabi 2021). There have been a number of recent studies developed to quantify the emissions of landfill gas using experimental and numerical approaches (Feng et al. 2020a; Ngusale et al. 2021). The field measurement of landfill gas emission includes a variety of different approaches, including the chamber techniques (Davidson et al. 2002; Izumoto et al. 2018; Wang et al. 2019; Zhan et al. 2020), eddy covariance techniques (Detto et al. 2011; Prajapati and Santos 2018), flux-gradient techniques (Zhao et al. 2019; Huang et al. 2022) and CH_4_ mixing ratio measurements (Dlugokencky et al. 2009).

Among the numerous techniques available to measure methane emissions from landfills, the static chamber method is the most popular due to its simplicity and relatively low cost (Senevirathna et al. 2007; Farkas et al. 2011; Maier and Schack-Kirchner 2014; Zhan et al. 2020; Yilmaz et al. 2021). For example, Haro et al. (2019) used the static chamber to assess the CH_4_ and CO_2_ surface emissions from Polesgo's landfill. The static chamber method combined with a laser methane detector and a biogas analyzer was applied to investigate the landfill gas emissions and methane (CH_4_) oxidation rates in landfill covers (Zhan et al. 2020). Yilmaz et al. (2021) used the static flux chamber technique to investigate the transport mechanisms of landfill gas through various cover soils. Although the gas emission flux can be easily and directly calculated from the linear regression of the concentration–time curves by using the static chamber (Senevirathna et al. 2006; Venterea et al. 2020), the accumulation of landfill gas in the chamber on the soil cover may lead to a decrease of the vertical concentration gradient and an increase of radial concentration gradient in the shallow ground (Venterea et al. 2020). Such changes may cause an unfavorable deviation (e.g., 27.8% underestimation) between the back-calculated and the actual fluxes (Janssens et al. 2000; Pape et al. 2009; Ding et al. 2015). The static chamber techniques could be a more attractive alternative if the errors/deviations can be minimized by a better design or operation.

Many works have been focused on studying effects of various factors, which include properties of the cover soil, the size of chambers, wind and pressures effects, etc., on errors and gas transport via experiments (Perera et al. 2002; Senevirathna et al. 2006; Venterea and Baker 2008; Venterea et al. 2009; Pihlatie et al. 2013; Redeker et al. 2015). For example, a laboratory experiment was conducted by Christiansen et al. (2011) to investigate the effects of the chamber height, soil properties and gas mixture on the relative error of the emission flux. The results showed that fluxes of CH_4_ were underestimated by a factor of 2 when the landfill gases were not well mixed. Davidson et al. (2002) reviewed several concerns about uncertainties of chamber-based methods and proposed corresponding approaches (e.g., using brief measurement periods, properly sized chamber and unrestricted flow) to minimize these errors and biases. The test results from Winton and Richardson (2016) indicated that concentrations of CH_4_ were particularly sensitive to variations of air pressure. The experimental results reported by Pihlatie et al. (2013) demonstrated that relative errors can be minimized by using a larger chamber. However, Ding et al. (2015) claimed that only the height of chamber can effectively reduce relative errors instead of the cover area or size of chamber.

Current understanding of the relative error induced by using static chamber has been achieved mainly through extensive experimental studies in the last decade, while the developments in the theoretical investigation have been rather limited (Sahoo and Mayya 2010; Venterea 2013). In recent years, extensive efforts have been devoted to modeling multi-component gases transport in the cover systems (Fen 2014; Ng et al. 2015; Feng et al. 2020b; Zuo et al. 2020; Bian et al. 2021). However, estimation models for landfill gases transport in the static chamber are limited to either empirical or single component models (Livingston et al. 2005; Senevirathna et al. 2007; Sahoo and Mayya, 2010; Parkin et al., 2012). For example, the traditional advection–diffusion (AD) model was widely used for investigating gas emission into the chamber headspace (Webb and Pruess 2003; Sahoo and Mayya, 2010; Cotel et al., 2015). Webb and Pruess (2003) pointed out that AD model may accurately describe emission of gases from a limited sample set on a single landfill site although it may over predict fluxes of traced gas for a lower gas permeability (*k*_*g*_ < 10^–13^ m^2^). Perera et al. (2002), Senevirathna et al. (2007), Bian et al. (2020), Ng et al. (2015) and Bian et al. (2021) simulated the movement of multi-component gases by using a model based on Blanc’s law, which considered reactive processes and variations of diffusion coefficient as a function of gas concentrations. It is noted that Blanc’s model can be only applied for investigating the multi-component gas system in which the tracer gas is dilute (Hibi et al. 2009). However, the landfill gases are a multi-component mixture, and the composition and concentration can be very complex at different stages (He et al. 2012; Gutiérrez et al. 2017). Especially for static chambers, gases may accumulate in the system after the emplacement of static chamber, which may contribute to vertical and lateral migration of gases in the shallow area. These issues can result in big errors by using Blanc’s model. Under these circumstances, the dusty-gas model (DGM), which considers the interactions between different component gas and the relationship between the gas concentration and the flux, would be more appropriate to investigate multicomponent gas emissions from the landfill cover (Fen 2014; Zuo et al. 2020). Although DGM is widely used for investigating multi-component gases migration in soils (Hibi et al. 2009; Fen, 2014; Zuo et al. 2020), the application of DGM for evaluating gas emission from the landfill cover and transport in the static chamber system are relatively rare.

The aim of this study is to address the need for quantifying the relative errors induced by using a static chamber and determining the effects of soil properties, chamber deployment strategies and flux calculation schemes on the relative errors to help design a better or conservative static chamber system. This is accomplished by presenting a set of theoretical formulations for coupling multi-component gas migration and exchange at the interface of soil and air. The developed model is then applied to assess the field monitoring data obtained by the static chamber method at a large-scale high kitchen food content landfill site in Xi’an, Northwest China.

## Material and methods

### Site description

**Fig. 1:**
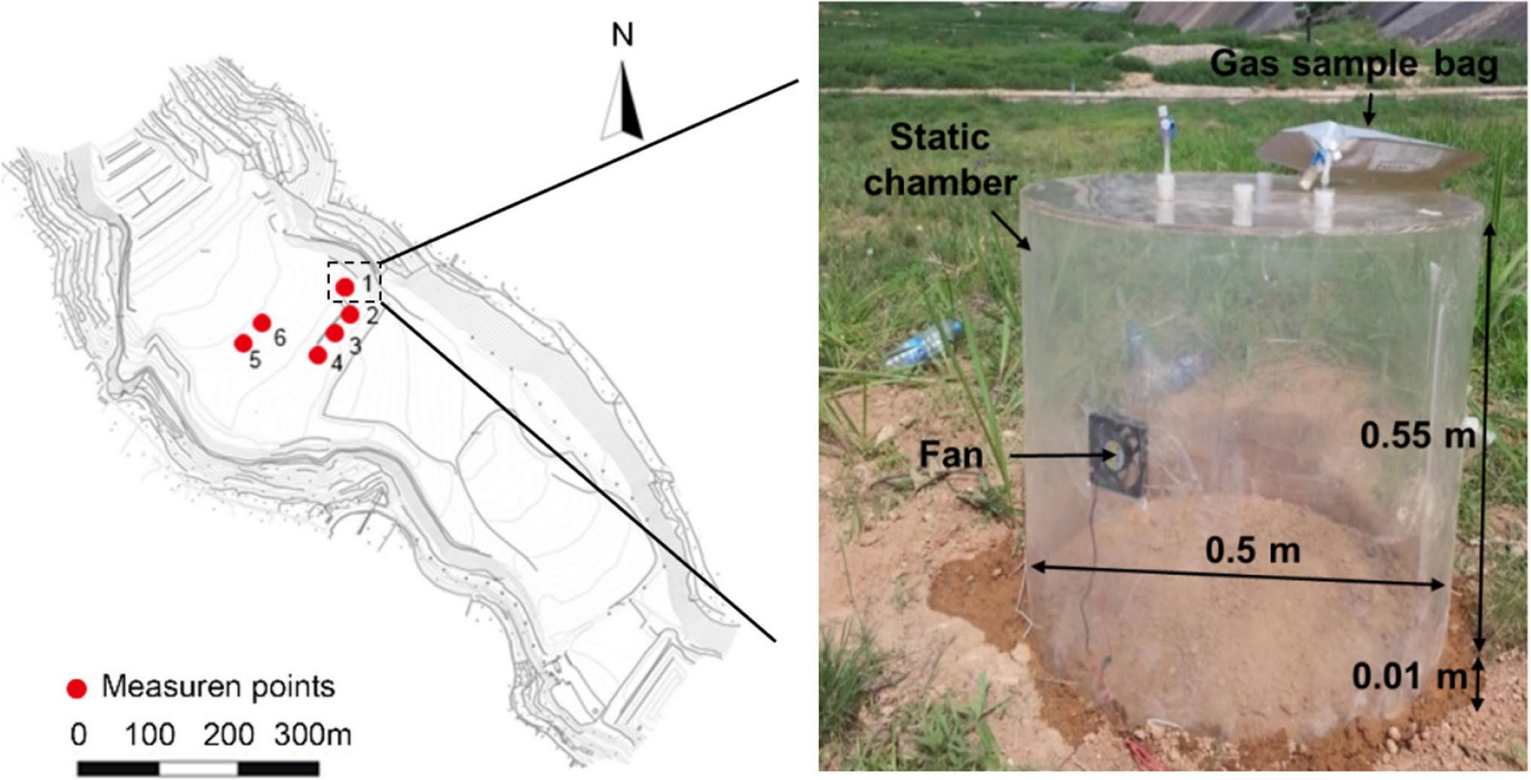
(**a**) Static chamber monitoring point at Jiangcungou landfill and (**b**) photograph of equipment applied in the field

The field test was conducted at the Jiangcungou landfill site on April 12th, 2015, which is located in the city of Xi’an in China. The area of the landfill is 7.34 × 10^5^ m^2^ with a capacity of 4.9 million m^3^ MSW disposal, which mainly includes kitchen waste (51.4%), paper (12%), wood (2%), textile (4.4%), plastic (14.8%) and ash (12.3%) (Shen et al., 2018). According to US EPA regulations, six monitored locations were regularly distributed on the surface of the temporary cover area (TVA) (Fig. 1), which was constituted by a 0.9-m-thick compacted loess layer (US EPA 2004). The distance among monitoring points was around 60 m. The MSW in TVA was less than 1 year. The dry density of the compacted loess at the top 0.3 m and 0.3–0.9 m is 1.3 g/cm^3^ and 1.45 g/m^3^, respectively. The porosity of loess ranges from 0.47 to 0.52. The gas diffusion coefficient in the loess cover obtained from the field test was 2.86 × 10^–13^ m^2^ (Zhan et al. 2016). The meteorological data, including wind speed, humidity, temperature, and atmospheric pressure during the field monitoring tests obtained from the local meteorological bureau are shown in Table 1.

**Table 1:** Meteorological values during field measurements

Date	Wind speed m/s	Humidity %	Atmospheric pressure hPa	Temperature ℃
12 April 2015	0.2–2.8	38.5–82.6	943–953.4	17.2–29.2

### Static chamber method

Figure 1b shows the static chamber used in the field. The chambers are made of plexiglass with a metal base. The radius and height of the chamber are 0.25 m and 0.55 m, respectively. The static chamber sidewall was equipped with a fan to mix the chamber headspace at a speed of 156 L/min (Christiansen et al. 2011). The fan was small, and the effect of the revolving speed of the fan on the monitored gas fluxes can be neglected (Gonzalez-Valencia et al. 2015; Tamminen et al. 2016). Tedlar bags and air pumps were used to collect LFG from selected locations. The flow rate of the air pump is 2 L/min. The capacity of the Tedlar bag for gas collection is 0.5 L, which accounts for 0.72% of the volume of the chamber. Therefore, it is reasonable to assume that volume change in the chamber induced by gas collection in the bag can be neglected (Wang et al., 2020). Both pumps (GP-2000) and Tedlar bags were provided by Huibin Instrument Company (Shanghai, China).

Firstly, the flexible pedestal was inserted into the soil with a depth of 0.01 m before the test. Once the chamber was placed at the sampling location, the groove in the metal base was filled with water to seal over the system during the test. All the valves were then shut off to allow gas to accumulate in the chamber. The sealing process can effectively prevent gas exchange between the chamber and the atmosphere (Wang et al. 2020). There was no air bubble in the water observed during the test, which indicates that the seal was good. The landfill gas was sampled every 10 min for 30 min. The concentration of CH_4_ in the air-bag was analyzed in the laboratory by the gas chromatograph GC9800, which has a high measurement accuracy for CH_4_ (e.g., within 0.1% error). 2 mL gas in the air-bag was manually injected into the gas chromatograph by the syringe. The gas chromatograph was equipped with a thermal conductivity detector (TCD). The carrier gas was H_2_. The temperature of the column, gasification chamber, and detector is 80 $^\circ{\rm C}$, 100 $^\circ{\rm C}$, and 120 $^\circ{\rm C}$ respectively.

The gas release flux is calculated by the gradient of concentration in the static chamber as follows (Rochette and Hutchinson, 2005):

1 $${N}_{measure}=\frac{{V}_{chamber}}{{A}_{chamber}}\times \frac{{C}_{t+\Delta t}-{C}_{t}}{\Delta t}=\frac{{V}_{chamber}}{{A}_{chamber}}\cdot k$$

where, *N*_measure_ (mol/m^2^/s) is the flux calculated by the concentration–time curve; *V*_chamber_ and *A*_chamber_ are the volume (m^3^) and base area (m^2^) of the chamber, respectively. *C*_*t*_ (mol/m^3^) and *C*_*t*+*Δt*_ represent the mole concentration of component *i* in the chamber at time* t* and *t* + Δ*t*, respectively; and *k* is the gradient of *C-t* curve. The slope (*k*) of the concentration–time curve was obtained by fitting the *C*-*t* curve.

## Mathematical model

**Fig. 2:**
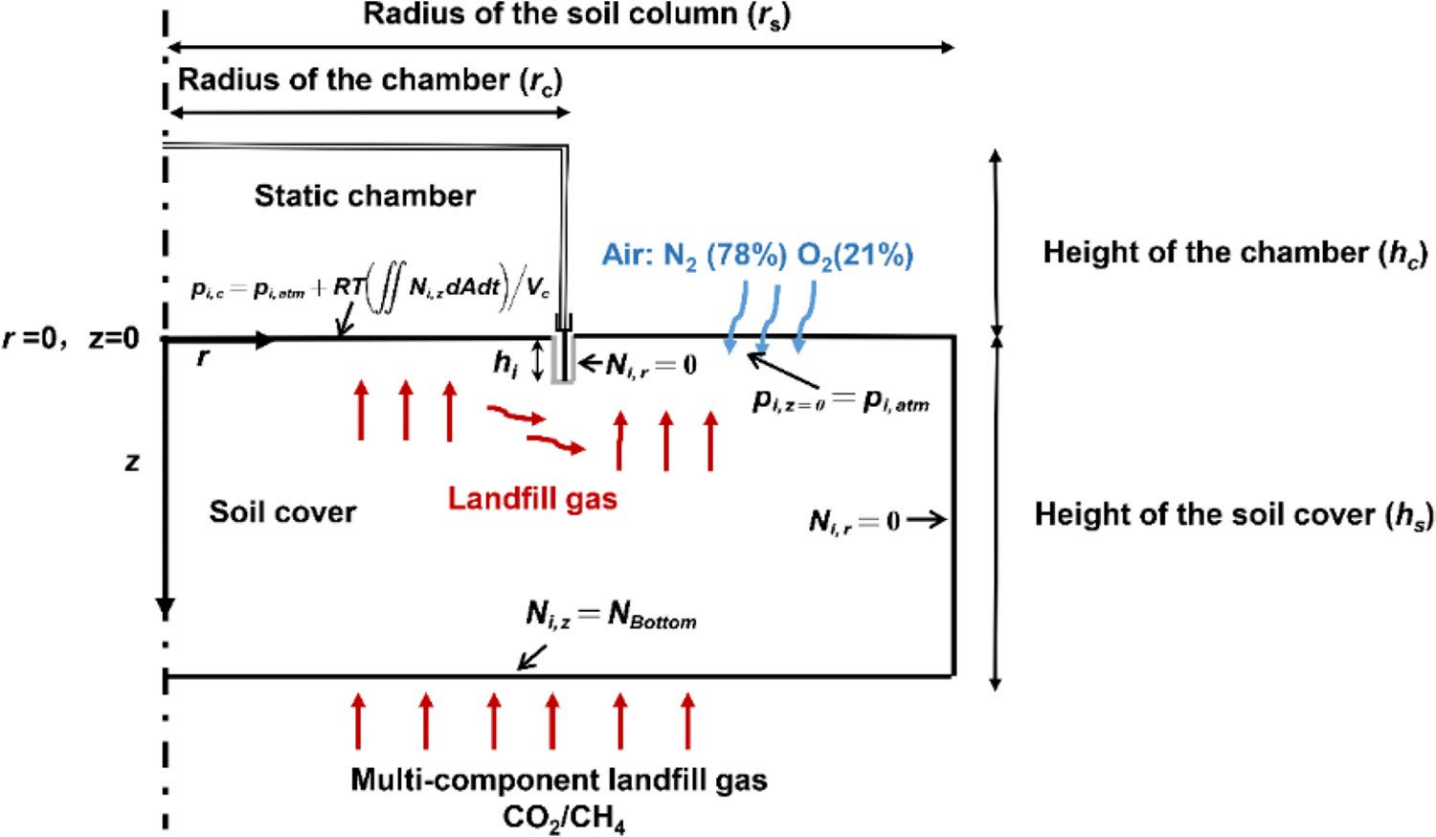
Schematic diagram of gas transport in cover soil with static chamber

The main assumptions for the proposed mathematical model are as follows: (1) gas is well mixed in the chamber; (2) the soil is homogeneous; (3) gas flux is distributed uniformly at the bottom of the cover soil; (4) effects of atmospheric pressure and temperature on gas transport are negligible; (5) gas transport in the soil reaches the steady state before the chamber is deployed over the soil. The schematic of gas transport in the cover soil with the static chamber is shown in Fig. 2.

### Governing equation

The theoretical formulation for two-dimensional multi-component gas transport in the cover system was given by (Bear 1972):

2 $${\theta }_{\mathrm{g}}\times \frac{1}{RT}\frac{{\partial p}_{i}}{\partial t}+\frac{1}{r}{N}_{i,r}+\frac{\partial {N}_{i,r}}{\partial r}+\frac{\partial {N}_{i,z}}{\partial z}={\rho }_{d}{r}_{i}$$

where *θ*_*g*_ is the air volume ratio; *z* and *r* (m) are the depth and radius, respectively; *p*_*i*_ (mol/m^3^) is the partial pressure of component *i*; *r*_*i*_ (mol/kg/s) represents the reaction rate of component *i*; *ρ*_*d*_ (kg/m^3^) is the dry bulk density of the soil; *N*_*i,z*_ and *N*_*i, r*_ (mol/m^2^/s) are the flux of component *i* in the axial and radial direction, respectively.

The total flux in direction *s* (include *z* or *r*) is given by (Mason and Malinauskas 1983):

3 $${N}_{i,s}={N}_{i,s}^{V}+{N}_{i,s}^{D}$$

where, *N*_*i,s*_^*D*^ is diffusion flux and *N*_*i,s*_^*V*^ is advection flux.

The advection flux can be derived based on Darcy’s law (Ho and Webb 2006)

4 $${N}_{i,s}^{V}=-\frac{{p}_{i}}{RT}\times \frac{{k}_{\mathrm{g}}}{\mu }\cdot \frac{dp}{ds}$$

where, *k*_*g*_ (m^2^) is the gas permeability; *μ* (Pa∙s) is the gas viscosity; *R* (m^3^/Pa·K/mol) is the ideal gas constant; *T* (K) is the absolute temperature; *p* is the total gas pressure.

The relationship between diffusion flux and partial pressure can be described by the Dust Gas Model (DGM) (Mason and Malinauskas 1983):

5 $$\begin{array}{cc}\sum_{j=1,j\ne i}^{n}\frac{{p}_{i}{N}_{j,s}^{D}-{p}_{j}{N}_{i,s}^{D}}{{\tau D}_{ij}p}-\frac{{N}_{i,s}^{D}}{{D}_{i}^{K}}=\frac{1}{RT}\cdot \frac{{\partial p}_{i}}{\partial s}& i=1,2,\cdots ,n\end{array}$$

where, *τ* is the tortuosity coefficient for the material; *D*_*ij*_ (m^2^/s) is the ordinary diffusion coefficient between components *i* and *j*; *D*^*K*^_*i*_ (m^2^/s) is the Knudsen diffusion coefficient for component *i*.

The Knudsen diffusion coefficient can be calculated by (Moldrup et al. 2000):

6 $${D}_{i}^{K}=\frac{{k}_{rg}k}{{\mu }_{i}}5.57{\left(k\cdot {k}_{rg}\right)}^{-0.24}\frac{{\mu }_{i}}{{\mu }_{air}}$$

The simplified Blanc’s model was also widely used to calculate the diffusion flux (Reid et al. 1987).

7 $${N}_{i,Blanc}^{D}=-{\tau D}_{i}\frac{\partial {C}_{i}}{\partial z}$$

where, the diffusion coefficient for the component *i* (*D*_*i*_) is given by:

8 $${D}_{i}=\frac{1}{\tau \sum_{\begin{array}{c}j=1\\ j\ne i\end{array}}^{n}\frac{{x}_{j}}{{D}_{ij}}}$$

where, *x*_*i*_ is the molar fraction of component *i*. *x*_*i*_ can be written as

9 $${x}_{i}={~}^{{C}_{i}}\!\left/ \!{~}_{\sum_{i=1}^{n}{C}_{i}}\right.=\frac{{p}_{i}}{p}$$

The reaction rate of methane (*r*_*CH4*_) is given by the following equation (Abichou et al. 2011):

10 $${r}_{{CH}_{4}}=\frac{{V}_{max}{x}_{{CH}_{4}}\cdot {x}_{{O}_{2}}}{\left({k}_{{CH}_{4}}+{x}_{{CH}_{4}}\right) \left({k}_{{O}_{2}}+{x}_{{O}_{2}}\right)}$$

where, *V*_*max*_ (mol/kg/s) is the maximum reaction rate of methane oxidation; *k*_CH4_ and *k*_O2_ are the half-saturation constant of CH_4_ and O_2_, respectively.

The reaction rate of CO_2_ and O_2_ can be given by (De Visscher and Cleemput 2003)

11 $${r}_{{CO}_{2}}=-{0.5r}_{{CH}_{4}}$$

12 $${r}_{{O}_{2}}=-{1.5r}_{{CH}_{4}}$$

By substituting the variable expressions in Eqs. (3), (4), (5), and (10) into Eq. (2), four linearly independent partial differential equations (CH_4_, CO_2_, O_2_, and N_2_) based on the DGM model with four components can be obtained as follows:

13 $$\begin{array}{c}\frac{{\theta }_{\mathrm{g}}}{RT}\times \frac{\partial }{\partial t}\left[\begin{array}{c}{p}_{1}\\ {p}_{2}\\ \begin{array}{c}\cdots \\ {p}_{n}\end{array}\end{array}\right]+\frac{1}{r}\left({\left({\varvec{B}}\right)}^{-1}\frac{1}{RT}\frac{\partial }{\partial r}\left[\begin{array}{c}{p}_{1}\\ {p}_{2}\\ \begin{array}{c}\cdots \\ {p}_{n}\end{array}\end{array}\right]+\frac{{k}_{rg}{k}_{i}}{RT\mu }\left(\frac{\partial p}{\partial r}\right)\left[\begin{array}{c}{p}_{1}\\ {p}_{2}\\ \begin{array}{c}\cdots \\ {p}_{n}\end{array}\end{array}\right]\right)\\ +\frac{\partial }{\partial r}\left({\left({\varvec{B}}\right)}^{-1}\frac{1}{RT}\frac{\partial }{\partial r}\left[\begin{array}{c}{p}_{1}\\ {p}_{2}\\ \begin{array}{c}\cdots \\ {p}_{n}\end{array}\end{array}\right]+\frac{{k}_{rg}{k}_{i}}{RT\mu }\left(\frac{\partial p}{\partial r}\right)\left[\begin{array}{c}{p}_{1}\\ {p}_{2}\\ \begin{array}{c}\cdots \\ {p}_{n}\end{array}\end{array}\right]\right)\\ +\frac{\partial }{\partial z}\left({\left({\varvec{B}}\right)}^{-1}\frac{1}{RT}\frac{\partial }{\partial z}\left[\begin{array}{c}{p}_{1}\\ {p}_{2}\\ \begin{array}{c}\cdots \\ {p}_{n}\end{array}\end{array}\right]+\frac{{k}_{rg}{k}_{i}}{RT\mu }\left(\frac{\partial p}{\partial z}\right)\left[\begin{array}{c}{p}_{1}\\ {p}_{2}\\ \begin{array}{c}\cdots \\ {p}_{n}\end{array}\end{array}\right]\right)=\left[\begin{array}{c}{r}_{1}\\ {r}_{2}\\ \begin{array}{c}\cdots \\ {r}_{n}\end{array}\end{array}\right]\times {\rho }_{d}\end{array}$$

and

14 $${\varvec{B}}=\left[\begin{array}{ccc}{B}_{11}& {B}_{12}& \begin{array}{cc}\cdots & {B}_{1n}\end{array}\\ {B}_{21}& {B}_{22}& \begin{array}{cc}\cdots & {B}_{2n}\end{array}\\ \begin{array}{c}\cdots \\ {B}_{n1}\end{array}& \begin{array}{c}\cdots \\ {B}_{n2}\end{array}& \begin{array}{c}\begin{array}{cc}\cdots & \cdots \end{array}\\ \begin{array}{cc}\cdots & {B}_{nn}\end{array}\end{array}\end{array}\right]$$

15 $${B}_{ii}=\frac{1}{{D}_{iM}}+\sum_{\begin{array}{c}j=1\\ j\ne i\end{array}}^{n}\frac{{p}_{j}}{{p\tau D}_{ij}}$$

16 $${{\varvec{B}}}_{ij\left(i\ne j\right)}=-\frac{{p}_{i}}{{p\tau D}_{ij}}$$

The surface flux without chamber is referred to as the reference flux. After the installation of chambers, the flux entering the chamber may be smaller than the real gas flux without chambers (called reference flux in the following section) due to the accumulation of gas concentration in the static chamber. The relative error between the flux entering the chamber and the reference flux is given by

17 $$\varepsilon =\frac{{N}_{ref}-{N}_{measure}}{{N}_{ref}}\times 100\%$$

where, *ε* is the relative error of flux; *N*_measure_ (mol/m^2^/s) is the flux entering the chamber; and *N*_ref_ (mol/m^2^/s) is the referenced emission flux without using the chamber.

### Boundary and initial conditions

#### Boundary conditions

At the surface of the soil outside the chamber, partial pressure for each component is considered as the atmospheric condition. The top boundary condition outside the chamber is:

18 $$p_{i,z=0}=p_{i,atm}=x_{i,atm}\cdot p_{atm}\;\;\;\;\;\;\;\;\;\;\;\;\;\;z=0,t\geq0$$

where, *x*_*i*,atm_ is the atmospheric mole fraction of component *i*; and *p*_*atm*_ is the absolute atmospheric pressure.

Gas concentrations in the chamber are assumed to be homogeneous after the chamber is deployed. It is assumed that the gases released from the soil surface mix well in the static chamber (see assumption 1). Thus, the top boundary condition inside the chamber is:

19 $$\begin{array}{cc}{p}_{i,chamber}={p}_{i,atm}+\frac{RT\iint {N}_{i,z}dAdt}{{V}_{chamber}}& r\le {r}_{c},z=0,t\ge 0\end{array}$$

where, the second term on the right side of Eq. (19) is the concentration of gases entering the static chamber when the chamber was deployed. *N*_*i,z*_ is the surface flux of component *i* at time *t*; *V*_*c*hamber_ is the volume of the static chamber and *r*_*c*_ is the radius of the chamber.

The bottom boundary is set as a constant flux since the gas generation rate at the bottom of the cover layer was assumed to be a constant:

20 $$N_{i,z}=N_{Bottom}\;\;\;\;\;\;\;\;\;\;\;\;\;\;\;\;\;\;\;\;\;\;\;z=-L,\;t\geq0$$

It should be noted that the bottom fluxes of CH_4_ and CO_2_, which represent the gas generation from the degradation of MSW, are constant. The fluxes of O_2_ and N_2_ are neglected at the bottom of the soil cover.

The inner wall of the insertion part of the chamber is impermeable. The horizontal flux at the inner wall of the chamber is assumed to be 0 (Sahoo and Mayya 2010):

21 $$\begin{array}{cc}N_{i,r}=0\;\;\;\;\;\;\;\;\;\;\;&r=r_c\end{array}$$

where, *N*_*i,r*_ is the radial flux of component *i*.

#### The initial conditions

Initial conditions for gas migration in the chamber were obtained from the steady-state model as the gas transport in the landfill cover was assumed to reach the steady-state before the placement of static chamber (see assumption 5). The steady-state concentration profiles of gases were obtained by using Eq. (13). In the steady-state model, the top boundary is assumed to be

22 $$p_{i,\;t=0}=p_{i,\;atm}\;\;\;\;\;\;\;\;\;\;\;\;t=0$$

A steady-state concentration profile of gas can be obtained by solving Eq. (13) combined with the boundary conditions (Eqns. 18 - 21). This concentration profile was then adopted as the initial concentration for the transient transport of landfill gas with the static chamber deployed.

Given the specific boundary condition in the “Boundary and initial conditions” section and the parameter values in Table 2, Eq. (13) can be solved using the PDE module in the finite element software, COMSOL Multiphysics software 5.1 (COMSOL, 2014). The convergence of the model was validated by using systematic mesh refinement to its geometry until grid-independent results were obtained. The mesh of the 2D model’s geometry was in the range of 3 × 10^–4^ -0.067 m.

**Table 2:** Parameters used in the comparison of experimental results and numerical model

Parameter			Perera et al. (2002)			Senevirathna et al. (2007)	
						Compost	Soil
Depth (m)			0–0.8			0–0.13	0.13–0.26
Horizontal permeability, *k*_gr_ (m^2^)			5 × 10^–10^			1 × 10^–13^	1 × 10^–12^
Vertical permeability, *k*_gz_ (m^2^)			1 × 10^–10^			1 × 10^–14^	1 × 10^–13^
Degree of saturation, *S*			0.25			0.10	0
Porosity, *n*			0.38			0.83	0.33
Air volume ratio, *θ*_g_			0.285			0.75	0.33
Tortuosity coefficient, τ			0.13			0.0625	0.1895
maximum reaction rate of methane oxidation, *V*_max_ (mol/kg/s)			0			7.7 × 10^–9^	0
Flux at the bottom boundary, *N*_bottom_ (mol/m^2^/s)			CO_2_: 5.2 × 10^–5^			CH_4_: 2.17 × 10^–4^ CO_2_: 7.89 × 10^–5^	
Size of chamber (m)				ID	H	ID: 0.1016 H: 0.1016	
		Small		0.1	0.05		
		medium		0.2	0.12		
		Large		0.25	0.16		
D_ij_ (m^2^/s ^a^)	_N2-__O2_	2.083 × 10^−^.^5^			_O2 -CO2_		1.635 × 10^–5^
	_N2__-CO2_	1.649 × 10^–5^			_O2—CH4_		2.263 × 10^–5^
	_N2__-CH4_	2.137 × 10^–5^			_CO2-CH4_		1.705 × 10^–5^

^a^Thorstenson and Pollock (1989)

## Validation of the proposed model

A series of experimental works on the transient surface emission of landfill gas by Senevirathna et al. (2007) and Perera et al. (2002) is used to validate the current numerical model. Perera et al. (2002) studied the effects of chamber size on the emission of CO_2_, while Senevirathna et al. (2007) mainly focused on investigating the variations of multi-components gases concentration in the chamber. The relative parameter values used for the simulations are shown in Table 2.

**Fig. 3:**
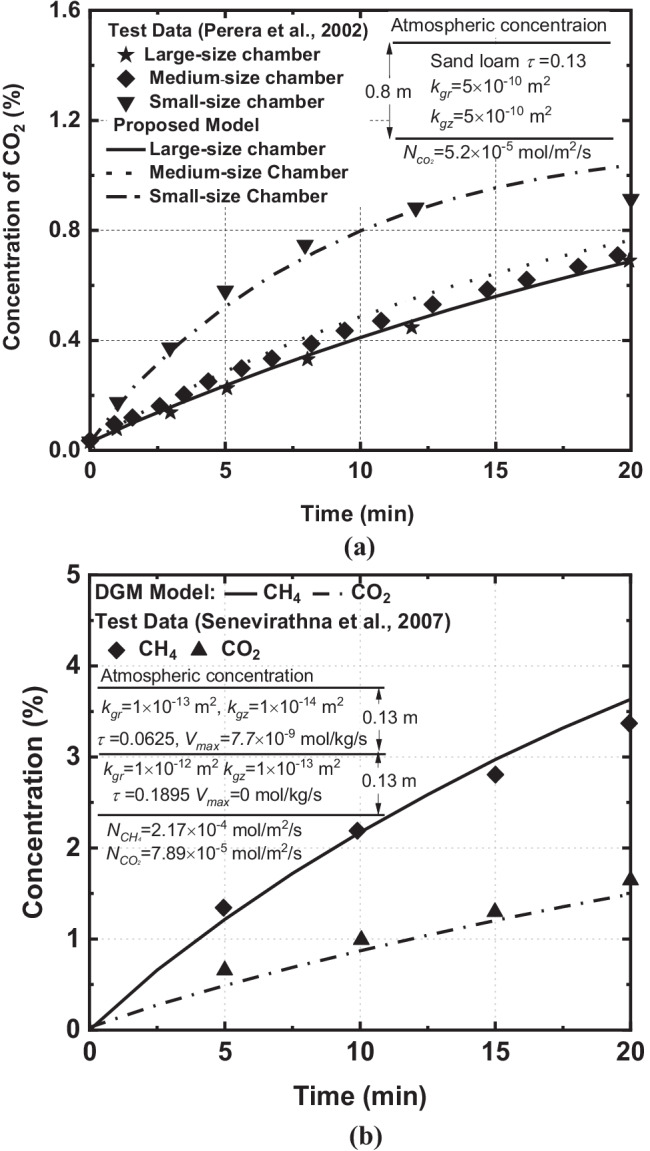
Gas concentration in the chamber over time: (**a**) the concentrations of CO_2_ under different sizes of chamber (Perera et al. 2002) and (**b**) multi-component landfill gas release from the landfill cover system (Senevirathna et al., 2007)

The inner diameter and height of the soil column used by Perera et al. (2002) were 0.45 m and 0.8 m, respectively. Three different sizes of chambers (e.g., 0.1 m × 0.05 m, 0.2 m × 0.12 m and 0.25 m × 0.16 m) were used to study the effects of chamber size on the emission of landfill gas. The bottom flux of CO_2_ was 199 g/m^2^/d (i.e., 5.2 × 10^–5^ mol/m^2^/s). Figure 3a shows the comparison of transient CO_2_ concentration between experimental data and numerical results with the different sizes of chambers. It can be seen that the CO_2_ concentrations predicted by the present model have good agreement with the experimental data. The experimental results demonstrated that increasing chamber size may lead to a fast increase of gas concentration in the first 15 min while the increasing rate was reduced in the last 5 min. This trend is correctly captured by the current model.

Multi-component gas (e.g., CH_4_, CO_2_, O_2_, and N_2_) transport in the landfill cover soils and the chamber was experimentally studied by Senevirathna et al. (2007). The thickness of the cover layer was 0.26 m. It consists of the top organic soil (0–0.13 m) and the bottom composting layer (0.13–0.25 m). The diameter and height of the static chamber were both 0.1016 m. The bottom fluxes for CH_4_ and CO_2_ were both 300 g/m^2^/d. The gas concentration at the top boundary was atmospheric concentration (i.e., *P*_*atm*_ = 101 kPa, *x*_CH4_ = 0%, *x*CO_2_ = 0.03%, *x*N_2_ = 78.5%, *x*O_2_ = 21.5%). Figure 3b shows the comparison of the evolution of CH_4_ and CO_2_ concentrations between the experimental data and the results obtained by the model. There is a good agreement between the present model and experimental data. The validations presented above provide confidence that the developed numerical model can be used to investigate the performance of multi-component gas transport in the cover system and static chamber.

These observations encourage further numerical study and quantification of the relative errors under different conditions (e.g., the effects of bottom flux condition, the insertion depth, and size of the chamber).

## Results and discussions

In this section, the significance of developments of DGM model for investigating multi-component gases transport in soil covered by the static chamber will be first illustrated by the comparison with the Blanc’s model. The effects of the key factors, including the size of the chamber, the insertion depth and pressure differential on the transport of gas in soil covered by the static chamber will be further investigated. The parameters used for simulations are shown in Table 3.

**Table 3:** main parameters for model calculation

Parameters		Values	Range
The bottom flux	*N*_*Bottom*_ (mol/m.^2^/s)	1 × 10^–5^	1 × 10^–9^-1 × 10^–3^
tortuosity coefficient	*τ*	0.1	0.001–0.9
gas permeability	*k*_*g*_ (m.^2^)	4 × 10^–12^	1 × 10^–19^-1 × 10^–9^
Height of the chamber	*h*_*c*_ (m)	0.55	0.275- 1.1
Radius of the chamber	*r*_*c*_ (m)	0.25	0.125–0.5
Insertion depth	*h*_*i*_ (m)	0.1	0.01–0.5

### Comparison of Blanc’s model and the proposed DGM model

Simulations were performed to examine the difference between the predictions of the present dusty gas model (DGM) (Eq. 5) and Blanc’s model (Eq. 7). It is noted that most of the existing numerical models for gas transport in the static chamber were developed based on Blanc’s law (Perera et al. 2002; Senevirathna et al. 2007; Bian et al. 2020, 2021; Ng et al. 2015). However, Blanc’s model may be only valid for investigating the multi-component gas system in which the tracer gas is dilute (Hibi et al. 2009). Gas accumulation induced by the installation of chambers may lead to inconsistent predicted results with field or experimental observation by using Blanc’s model. Therefore, from the analysis of field data and considering the limitations of Blanc’s model, DGM is introduced in this study to investigate multi-component gases transport in soil covered by the static chamber.

**Fig. 4:**
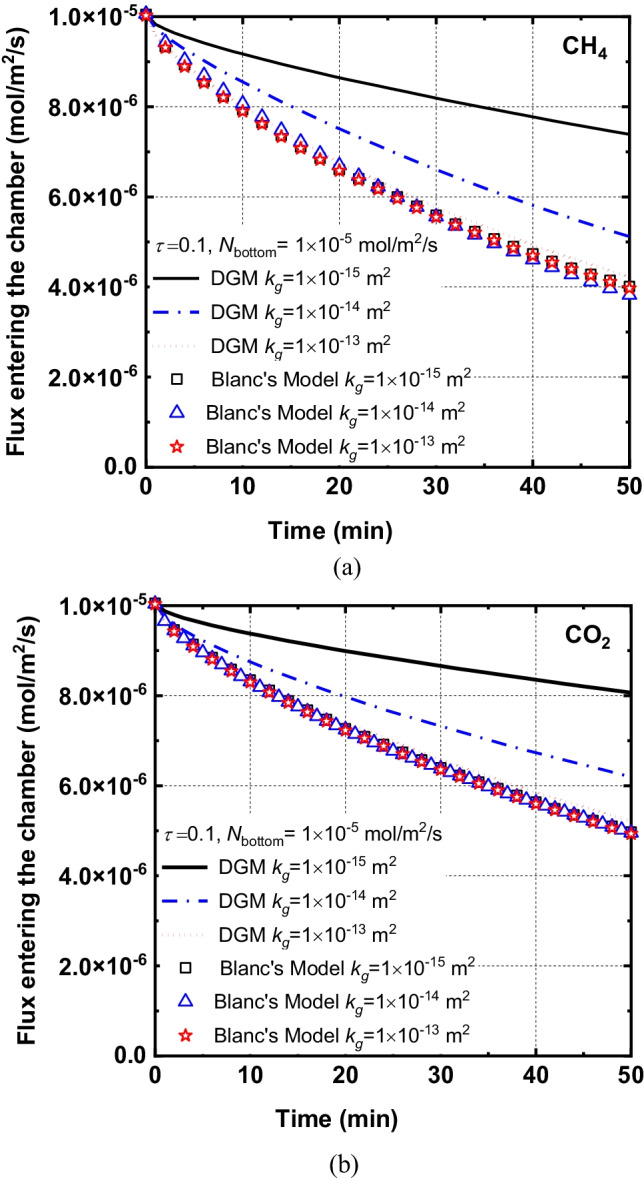
Flux of CH_4_ and CO_2_ entering the chamber calculated by the Blanc's model (*N*_Blanc’s_) and the DGM model (*N*_DGM_): (**a**) CH_4_ and (**b**) CO_2_

Comparisons of CH_4_ and CO_2_ fluxes obtained by Blanc’s model and DGM model were demonstrated in Fig. 4. Generally, emission fluxes of CH_4_ and CO_2_ from the covered soil into the chamber increased with time increasing. It is noted that fluxes predicted by DGM model and Blanc’s model are very close for the case with *k*_*g*_ = 10^–13^ m^2^ while differences increase with the reduction of the permeability. For example, the flux of CH_4_ obtained by DGM model was 1.9 times greater than that of Blanc’s model for *k*_*g*_ = 10^–15^ m^2^ at *t* = 50 min. Furthermore, the effects of the permeability on emission fluxes of gases (e.g., CH_4_ and CO_2_) are negligible for Blanc’s model. There are two reasons for the difference obtained by DGM and Blanc’s model. First, decreasing the permeability of cover soil can reduce the advection flux of gases(Zuo et al. 2020). As a result, diffusion mechanisms may play a dominant role in governing gases transport. The interaction among gases (e.g., collision) is enhanced, which can be captured by DGM model (Krishna and Wesselingh 1997). Secondly, a lower permeability can enhance the Knudsen diffusion process, which leads to an increase in concentration gradients, while it is not considered in Blanc’s model. Moreover, the Knudsen diffusion process may contribute to the migration of gas when the gas permeability is less than 10^–13^ m^2^. Similar trends were reported by Fen (2014) that the Knudsen diffusion process may affect gas transport in soils with low permeability.

The above results indicate Blanc’s model cannot be applied to a multi-component gas system with a wide range of gas concentration and low gas permeability soil (e.g., *k*_*g*_ < 10^–13^ m^2^). Therefore, developments of DGM model are necessary to improve the predicted capacity of numerical models for studying multi-component gas transport in the cover soil and the static chamber even for the case with low permeability cover.

### Effects of chamber sizes on the relative error (ε)

**Fig. 5:**
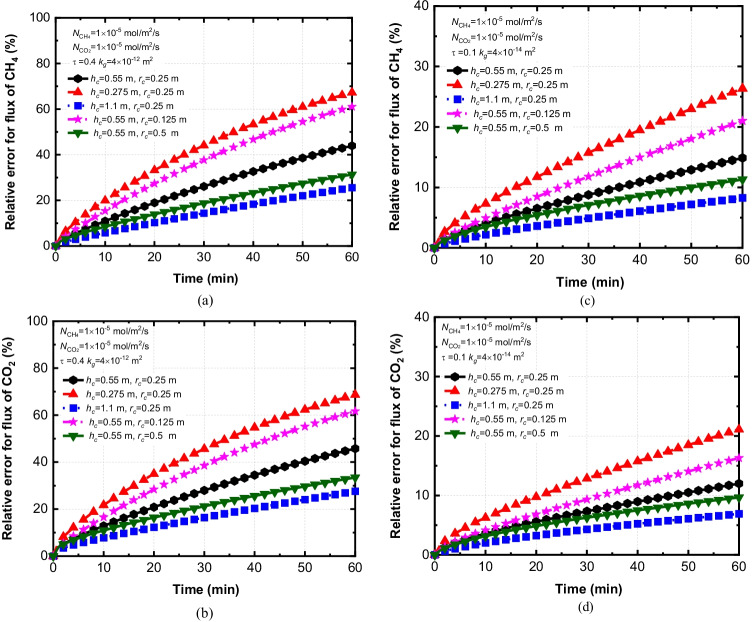
The effect of *h*_*c*_ and *r*_*c*_ on the relative error for (**a**) CH_4_ with *τ* = 0.4, *k*_*g*_ = 4 × 10^–12^ m^2^, (**b**) CO_2_ with *τ* = 0.4, *k*_*g*_ = 4 × 10^–12^ m^2^, (**c**) CH_4_ with *τ* = 0.1, *k*_*g*_ = 4 × 10^–14^ m^2^ and (**d**) CO_2_ with *τ* = 0.1, *k*_*g*_ = 4 × 10^–14^ m.^2^

Figure 5 shows the influence of the radius (*r*_*c*_) and height (*h*_*c*_) of the static chamber on the relative errors (*ε*). The chamber size with *h*_*c*_ = 0.55 m and *r*_*c*_ = 0.25 m used in the field test is considered as a reference case. It can be found that the relative error increases with the deployment time increasing. The predicted relative error for the flux of CH_4_ and CO_2_ showed similar trends. Generally, increasing the size of static chamber, including the height and the radius, can reduce the relative error for the flux of CH_4_ and CO_2_. Increasing *h*_*c*_ from 0.55 m to 1.1 m can reduce the relative errors of CH_4_ from 44 to 25% at *t* = 60 min for *τ* = 0.4, *k*_*g*_ = 4 × 10^–12^ m^2^. Similarly, when the radius of the chamber increases from 0.25 m to 0.5 m, the relative error of CH_4_ is decreased by 13.1%. Similar observations were reported by Pihlatie et al. (2013) that the underestimation of the flux decreased with increasing chamber size. However, Ding et al. (2015) reported a different conclusion that the relative error was only sensitive to the height of the chamber instead of the radius or volume of the chamber. The difference reported in existing literature may be attributed to characterizations of soils (e.g., tortuosity and permeability of soils). Figure 5a and c shows effects of chamber sizes on relative errors of CH_4_ under different soil characterizations, including cases with *τ* = 0.4, *k*_*g*_ = 4 × 10^–12^ m^2^ and *τ* = 0.1, *k*_*g*_ = 4 × 10^–14^ m^2^. Correspondingly, effects of chamber sizes on relative errors of CO_2_ under different soil characterizations are demonstrated in Fig. 5b and d. It can be seen that relative errors of CH_4_ and CO_2_ are less influenced by chamber sizes for the case with compacted soils (e.g., *τ* = 0.1, *k*_*g*_ = 4 × 10^–14^ m^2^). For example, decreasing the height of chamber for *τ* = 0.1and *k*_*g*_ = 4 × 10^–14^ m^2^ may increase relative errors of CH_4_ and CO_2_ by 11.5% and 9.1% than that of *τ* = 0.4 and *k*_*g*_ = 4 × 10^–12^ m^2^. However, reducing the radius of chamber for *τ* = 0.1and *k*_*g*_ = 4 × 10^–14^ m^2^ may only increase relative errors of CH_4_ and CO_2_ by 6.1% and 4.3% than that of *τ* = 0.4 and *k*_*g*_ = 4 × 10^–12^ m^2^. The above results indicate that relative errors are more sensitive to variations in chamber heights compared to changes in chamber radius when *τ* = 0.1 and *k*_*g*_ = 4 × 10^–14^ m^2^.

In conclusion, increasing the height as well as the radius of the chamber can be an effective approach to decrease the relative error for the case with greater gas permeability and diffusion coefficient (e.g., *τ* = 0.4, *k*_*g*_ = 4 × 10^–12^ m^2^). Additionally, shortening deployment times may improve the accuracy of the flux predictions.

### Effects of the insertion depth (h_i_) of chamber on the relative errors

**Fig. 6:**
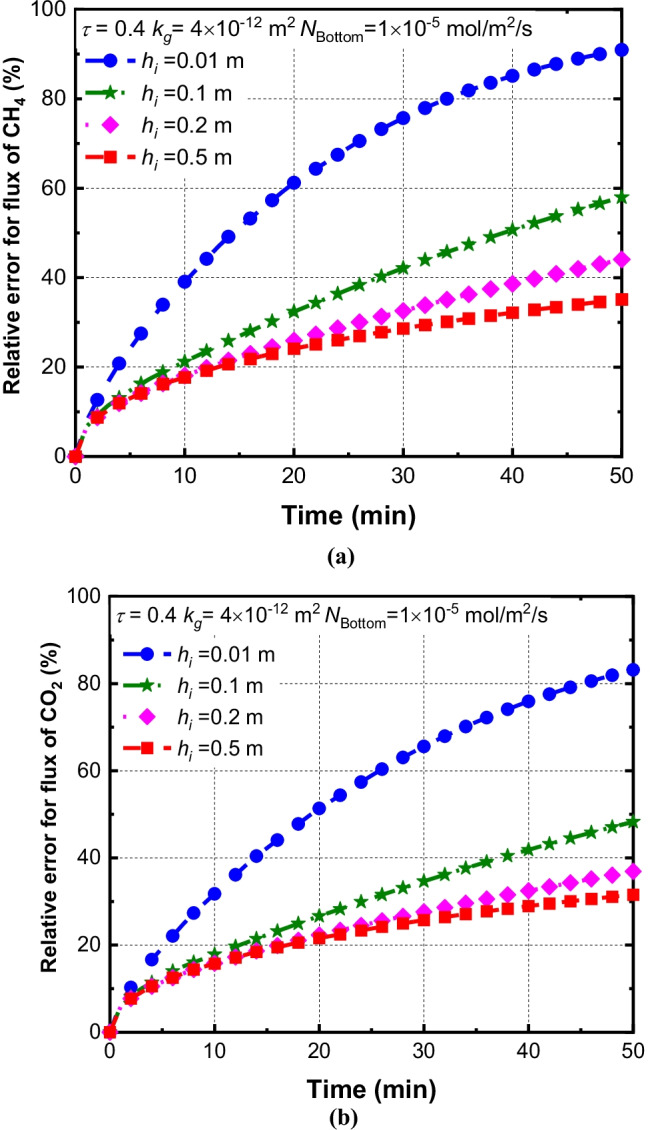
Effcts of insertion depth (*h*_*i*_) on relative errors of (**a**) CH_4_ and (**b**) CO_2_

Figure 6 shows the effects of the insertion depth on the relative errors of CH_4_ and CO_2_ fluxes. The insertion depth varies from 0.01 to 0.5 m. It can be seen that the relative errors of CH_4_ fluxes reduce with the insertion depth increasing. When the insertion depth increases from 0.01 m to 0.1 m, the relative error of CH_4_ and CO_2_ flux decreases from 94.4 to 64.1% and 83.2 to 48.2% at *t* = 50 min, respectively. It can be explained that increasing the insertion depth can prevent the horizontal transport of gas in the shallow area, which leads to the reduction of the deviations of the measured gas flux. However, the relative error for CH_4_ decreases slightly (e.g., 7.2%) when the insertion depth increases from 0.2 to 0.5 m. The result indicates that the horizontal gas transport is only significant for the case within the depth of 0.2 m. Additionally, it can be seen that the relative error of CH_4_ fluxes is larger than that of CO_2_. This may be explained by that CO_2_ has a larger molecular mass and samller diffusion coefficient compared to CH_4_, which leads to a lower the relative error of CO_2_.

**Fig. 7:**
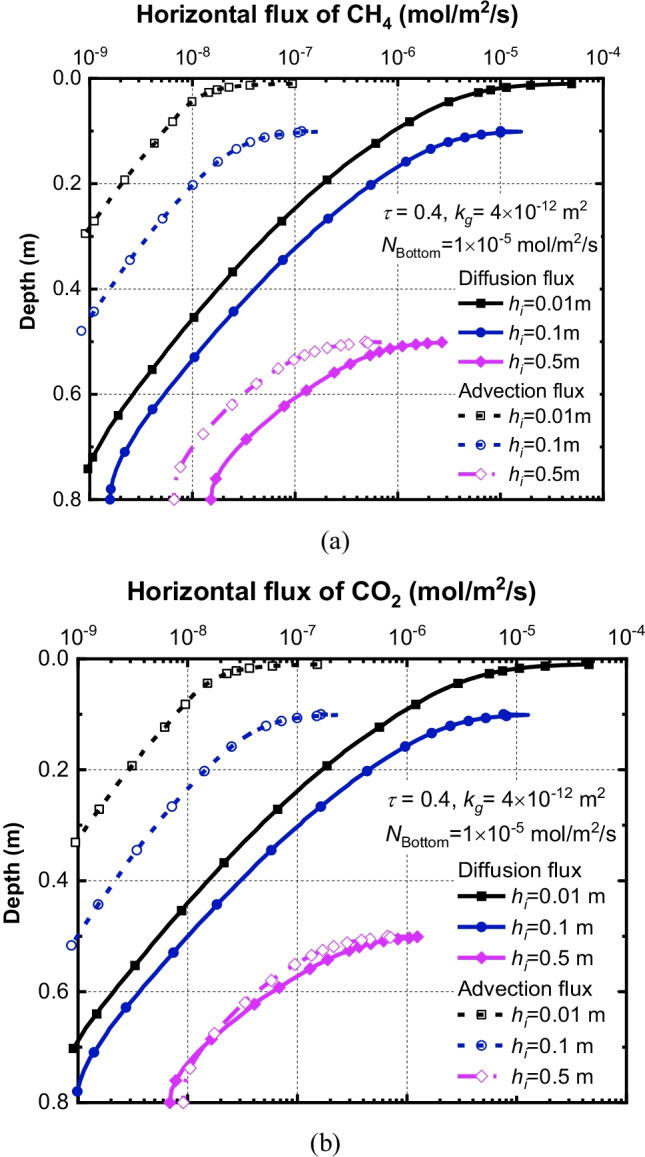
Effects of *h*_*i*_ on the horizontal flux of (**a**) CH_4_ and (**b**) CO_2_

In order to further explore the influence of insertion depth on the relative error, the horizontal advection fluxes and diffusion fluxes are plotted in Fig. 7. It is demontrated that the dominant mechanism for CH_4_ and CO_2_ transport in the horizontal direction in the system is diffusion. Figure 7 shows that horizontal advection fluxes of CH_4_ and CO_2_ are one order magnitude less than that of diffusion fluxes for the case with *h*_*i*_ = 0.01 and 0.1 m. This observation is consistent with phenomena reported by Bian et al. (2021) that the diffusion process played the main role in governing the transport of gases in the shallow area. Increasing insertion depth can impede the horizontal diffusion fluxes of gases. For example, increasing insertion depth from 0.01 m to 0.1 m results in a decrease in diffusion fluxes of CH_4_ and CO_2_ by a factor of 0.2 and 0.16, respectively. Therefore, a reduction of relative errors induced by increasing insertion depth can be attributed to the horizontal diffusion of gases (Fig. 7). These results demonstrated that lateral gas transport mainly occurs in the shallow area of the soil, due to the fact that the accumulation of concentration is within the depth of 0.1 m. The above results indicated that the insertion depth of the chamber should be greater than 0.1 m in field tests.

### Effects of pressure differential near the surface boundary on emission fluxes

In this section, effects of the pressure difference between the inside and outside the chamber at the top boundary on emission fluxes of CO_2_ and CH_4_ are quantified. Pressure differential (*∆P*_*in-out*_) may be driven by spatially and temporally variable winds and pressures after the installation of chambers (Xu et al. 2006; Liu et al. 2019). It is reported that the pressure is sensitive to variations of wind speed when the speed is above an certain value (e.g., 3 m/s) (Chi et al. 2013; Laemmel et al. 2017).The pressure differential may have a range from -0.1 kPa to 0.1 kPa (Gebert et al. 2011; Redeker et al. 2015). It should be noted that fluctuations of wind speed may also affect gas transport process from the cover soil to the air and mixing in the headspace (Ahmadi et al., 2021), which was not assessed in this study.

**Fig. 8:**
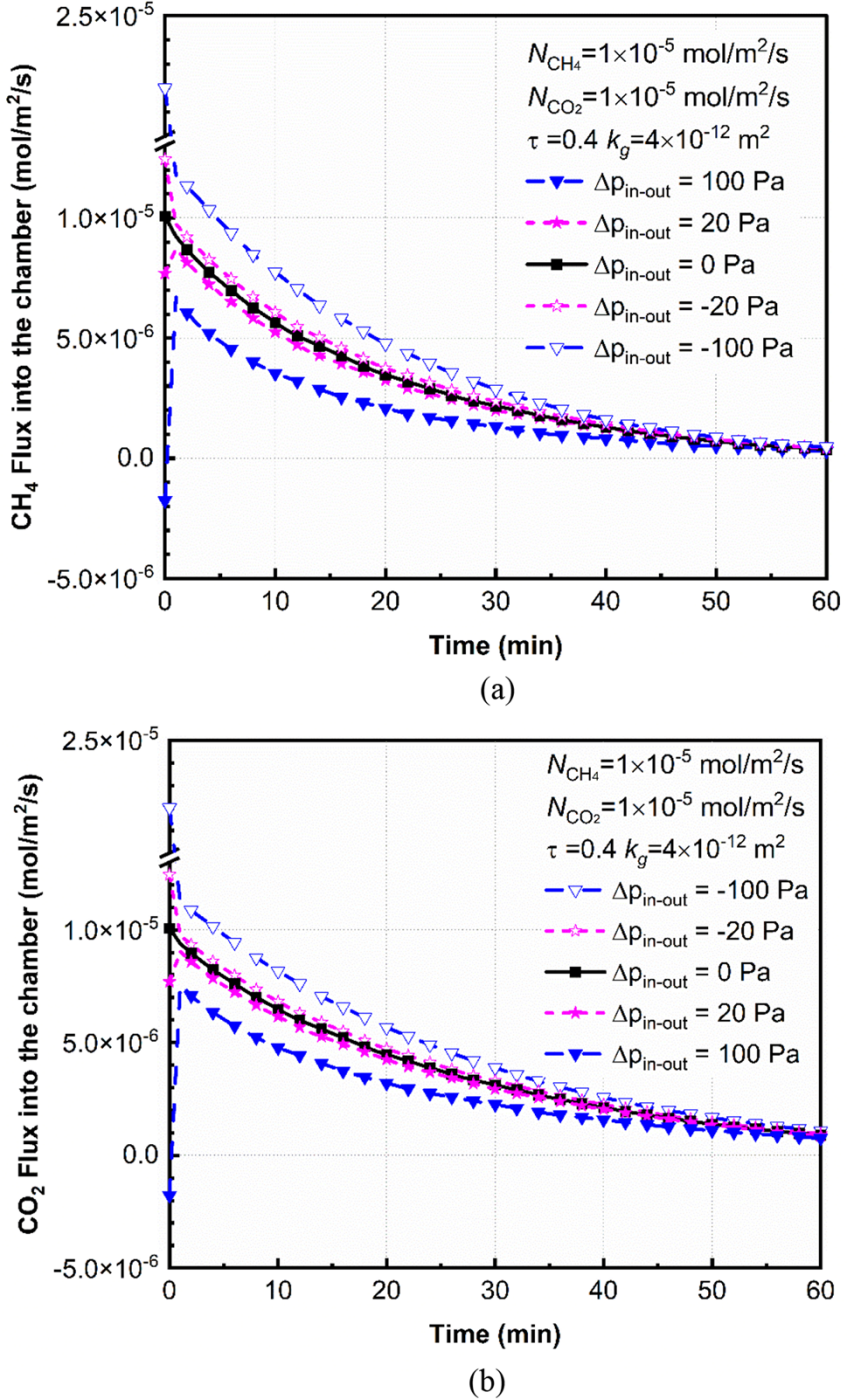
The effect of air pressure fluctuation on gas emission flux at the soil surface in the area covered by static chamber (**a**) CH_4_ and (**b**) CO_2_

Figure 8 shows the effects of *∆P*_*in-out*_ on surface-emission fluxes of CH_4_ and CO_2_ in the chamber. The positive pressure differential represents the case that pressure inside the chamber is larger than that outside the chamber. *∆P*_*in-out*_ = 0 is selected as the reference case to investigate effects of pressure differential on emission fluxes. We find that gas emission fluxes are greater than that of the reference case when the pressure differential (*∆P*_*in-out*_) is positive. For example, emission fluxes of CH_4_ and CO_2_ are decreased by 41% and 28% for *∆P*_*in-out*_ = 100 Pa compared to the reference case at *t* = 20 min. The positive pressure differential (*∆P*_*in-out*_ > 0) may cause a temporary increase in vertical pressure gradient and concentration differences of gases between the inside and outside chambers. As a result, gases can transport from the chamber to cover soils in the near-surface area, which leads to lower gas fluxes in the chamber. Additionally, effects of pressure differential are reduced with increasing deployment times. For example, the negligible difference may be found for different *∆P*_*in-out*_ when *t* > 50 min (see Fig. 8). This is due to the fact that although pressure differential can temporally affect the distribution of gas fluxes and concentrations, the pressure differential caused by the temporary pressure fluctuation in the top boundary may decrease with times increasing as the bottom boundary was set as a constant flux in the simulation (Eq. 20).

## Applications of the model in a field study

**Fig. 9:**
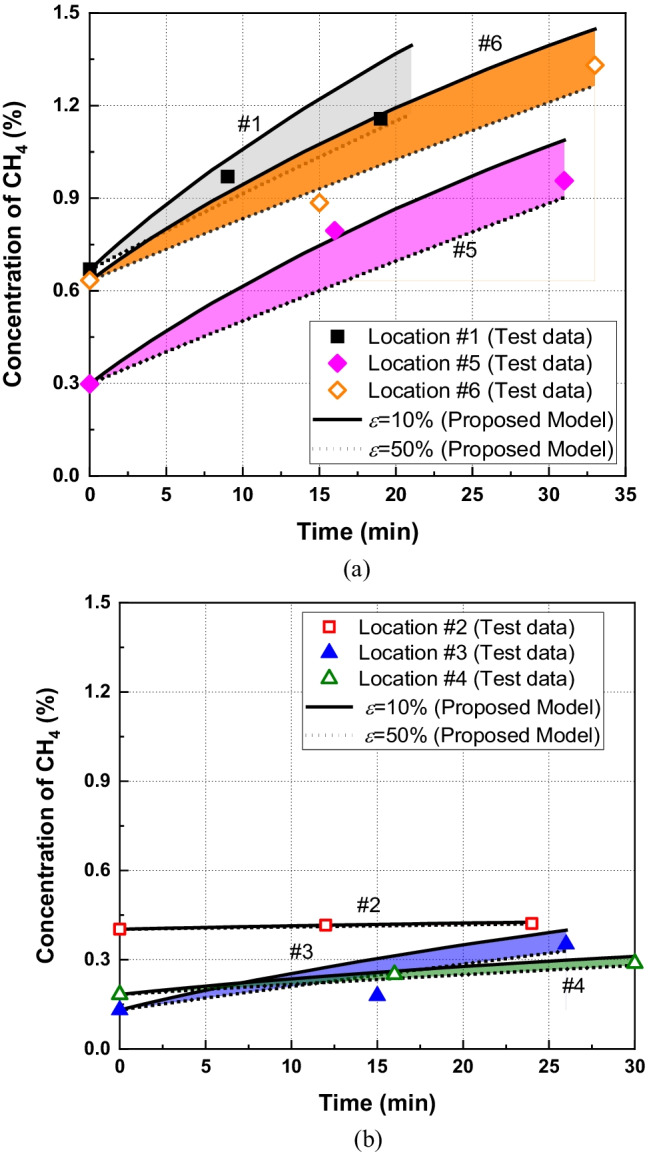
Comparison of CH_4_ concentration between the field data and numerical results

The concentrations of CH_4_ in the chamber at different field locations are shown in Fig. 9. The locations of the monitoring points are shown in Fig. 1 and were chosen according to US EPA (2004). The measured methane emission fluxes can be obtained by Eq. (1) based on the *C-t* data. The gas flux calculated by the linear regression method at different monitored locations is provided in Table 4. It can be seen that the gas flux ranges from 2.7 × 10^–6^ to 8.7 × 10^–5^ mol/m^2^/s, which is coincident with observations in the high-level kitchen waste content MSW in China (e.g., 4.69 × 10^–8^-6.25 × 10^–5^ mol/m^2^/s in Xiamen, 0–1.0 × 10^–3^ mol/m^2^/s in Beijing and 5.3 × 10^–6^ -2.5 × 10^–5^ mol/m^2^/s in Nanjing) (Wang et al. 2017; Li et al. 2020; Zhang et al. 2019). The dry density of the compacted loess at the top 0.3 m and 0.3–0.9 m is 1.3 g/cm^3^ and 1.45 g/m^3^, respectively. The porosity of loess ranges from 0.47 to 0.52. The gas diffusion coefficient in the loess cover obtained from the field test was 2.86 × 10^–13^ m^2^ (Zhan et al. 2016). The meteorological data, including wind speed, humidity, temperature, and atmospheric pressure, during the field monitoring tests obtained from the local meteorological bureau are shown in Table 1. It should be pointed out that extensive studies have demonstrated that the surface gas emission fluxes may exhibit prominent spatial and temporal variations as landfill gas emissions may be largely affected by waste age, cover types, components of wastes and landfill management strategies (Pierini et al. 2018; Wang et al. 2020; Duan et al. 2021). The organic content, porosity and water distribution in cover soils can affect the reaction rate and migration of gases in the system. Moreover, key experimental factors (e.g., temperature, wind speed and air pressure on the near surface of soils) may contribute to fluctuations of gas emission rate (Bian et al. 2019; Pinheiro et al. 2019; Zhan et al. 2020). It should be noted that the above factors are not considered in this study. This is considered as a limitation of the current study.

**Table 4:** Gas flux calculated by the linear regression method and the numerical model

Position	*N*_*measure*_ Linear regression mol/m.^2^/s	Assumed *ε* %	*N*_*ref*_ Numerical model mol/m.^2^/s	*R*.^2^ for numerical model	*τ*	*k*_*g*_ m.^2^
Location 1	8.76 × 10^–5^	30.0%	1.25 × 10^–4^	0.9934	0.4	1 × 10^–12^
Location 2	2.70 × 10^–6^	35.0%	4.15 × 10^–6^	0.9781	0.5	1 × 10^–11^
Location 3	2.81 × 10^–5^	6.7%	3.00 × 10^–5^	0.9176	0.1	1 × 10^–15^
Location 4	1.21 × 10^–5^	28.4%	1.69 × 10^–5^	0.9994	0.4	1 × 10^–14^
Location 5	7.33 × 10^–5^	35.0%	1.13 × 10^–4^	0.9824	0.5	1 × 10^–11^
Location 6	7.30 × 10^–5^	10.0%	8.11 × 10^–5^	0.9920	0.1	1 × 10^–14^

The maximum measured concentration of CH_4_ was in position 1 (called p_1_), which is then followed by p_6_, p_5_, p_2_, p_4_, and p_3_. It can be seen that the concentration of CH_4_ at p_2_, p_4_ and p_3_ is significantly lower than that observed at p_1_. A possible explanation for this might be that p_2_, p_4_ and p_3_ had lower CH_4_ emission fluxes. This resulted in less CH_4_ concentration observed in the chamber. Additionally, the rate of concentration increase was reduced with time increasing except for p_3_ and p_6_. It demonstrated that the emission of CH_4_ was attenuated by the chamber. The controversial trend observed at p_3_ and p_6_ might be attributed to the pressure fluctuations and the mass flow of soil air induced by the chamber placement (Takle et al. 2004; Bain et al. 2005).

The numerical results obtained by the present model are provided in Fig. 9. The gas components considered in the model are CH_4_, O_2_ and N_2_. The geometry of the model is the same as the chamber used in the field test with *h*_*c*_ = 0.55 m and *r*_*c*_ = 0.25 m. The top boundary was fixed as the gas concentrations in air. The bottom boundary was assumed to be a constant flux, which can be approximated by the referenced emission flux (*N*_*ref*_) given in Eq. (17). It can be seen that *N*_*ref*_ is dependent on the relative error (*ε*), which is largely affected by properties of soil (e.g., diffusivity and permeability). The Monte Carlo (MC) method, also known as statistical simulation method, was adopted in this section to carry out the statistical analysis of different parameters (e.g., gas permeability, diffusivity and tortuosity of the soil cover). The simulation starts with the characterizations of uncertain parameters. Appropriate probability distributions are used to model uncertainties in the input parameters. The field data reported by (Moldrup et al. 2000; Fujikawa and Miyazaki 2005; Fen 2006; Wickramarachchi et al. 2011; Garg et al. 2019; Li et al. 2019; Huang et al. 2020) showed that the mean of tortuosity and permeability ranges from 0.001 to 0.9 and 10^–19^ and 10^–9^ m^2^, respectively. Gas permeability and tortuosity are assumed to obey normal distribution in this section. Then, a large number of random samples (e.g., 1000) of the uncertain variables are generated from their respective probability distributions, followed by relative errors calculation using Eq. (17). Finally, a statistical analysis of the output is performed to estimate the range of relative errors induced by using static chambers.

**Fig. 10:**
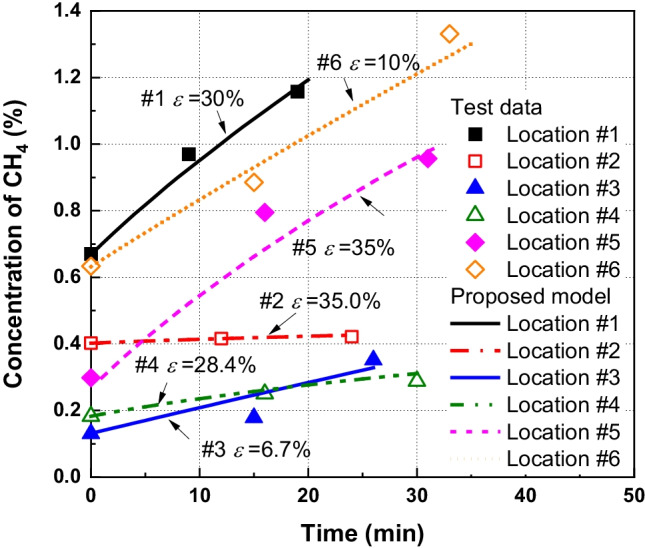
Comparison of CH_4_ concentration between the field data and numerical results with fitted relative errors

Figure 10 shows the predicted results obtained by the proposed model with *ε* ranging from 10 to 50%. It should be noted that a large relative error (e.g., *ε* = 50%) can be observed for the case with the high gas permeability and diffusion coefficient (e.g., *k*_*g*_ = 1.0 × 10^–11^ m^2^ and *τ* = 0.6); conversely, a small relative error (e.g., *ε* = 10%) represents the soil with the low gas permeability and diffusion coefficient (e.g., *k*_*g*_ = 1.0 × 10^–14^ m^2^ and *τ* = 0.1). It can be seen that the measured concentration of CH_4_ is almost within the ranges obtained by the numerical model. Figure 10 shows that the best fitted relative error for p_1_, p_2_, p_3_, p_4_, p_5_ and p_6_ is 30.0%, 35%, 6.7%, 28.4%, 35.0%, and 10.0%, respectively. All these fitted relative errors are in the range obtained by the Monte Carlo (e.g., 0.03–60%). The above results demonstrate that the proposed numerical model has the capacity to quantify the concentration of CH_4_.

## Conclusions

A field test of landfill gas emission is conducted by using the static chamber at one landfill site located in Xi’an, Northwest China. In order to investigate the reliability of the chamber method, a two-dimensional axisymmetric numerical model for multi-component gas transport in the soil and the static chamber is developed based on DGM. The proposed model is validated by the field data obtained in this study as well as a set of experimental data in the literature. The proposed model is used to investigate the effects of the insertion depth and size of the chamber on the relative errors.

A comparison of DGM model and Blanc’s method indicates that DGM model has the capacity to predict the gas transport under a wider range of permeability (*k*_g_ < 10^−14^ m). For example, the flux of CH_4_ obtained by DGM model was 1.9 times greater than that of Blanc’s model for *k*_*g*_ = 10^–15^ m^2^ at *t* = 50 min. This is due to the fact that DGM model can explain the interaction among gases (e.g., CH_4_, CO_2_, O_2_ and N_2_) and the Knudsen diffusion process while these mechanisms are not included in Blanc’s model.

The results showed that increasing the size of the chamber and shortening the deployments times may be effective ways to reduce relative errors of CH_4_ and CO_2_ fluxes. For example, increasing the radius of the chamber from 0.25 m to 0.5 m can lead to a reduction of 13.1% and 12.3% of the relative error for CH_4_ and CO_2_ fluxes. Additionally, the relative errors of CH_4_ fluxes reduce with the increase of insertion. The relative error of CH_4_ and CO_2_ flux decreases from 94.4% to 64.1% and 88.2% to 54.0% when the insertion depth increases from 0.01 m to 0.1 m. The reason is that increasing the insertion depth can prevent the horizontal transport of gas in the shallow area, which leads to the reduction of the deviations of the measured gas flux.

The developed multi-component landfill gas transport model is applied to assess the field monitoring data of CH_4_ fluxes obtained by the static chamber method. The Monte Carlo method was adopted to carry out the statistical analysis for quantifying the range of the relative errors for CH_4_ fluxes. The agreement of the measured field data and predicted results demonstrated that the proposed model has the capacity to quantify the emission of landfill gas from the landfill cover systems.

## Data Availability

Data are available from the authors upon reasonable request.

## Notation

*V*_chamber_: (M^3^) the volume of the chamber
*A*_chamber_: (M^2^) the base area of the chamber
*r*_*c*_: (M) the radius of the chamber
*h*_*c*_: (M) the height of the chamber
*C*_*t*_: (Mol/m^3^) the mole concentration of component *i* in the chamber at time *t*
*C*_*t*__+__*Δ*__*t*_: (Mol/m^3^) the mole concentration of component *i* in the chamber at time *t* + Δ*t*
*K*: The gradient of *C**-**t* curve
*θ*_*g*_: The air volume ratio
*S*: Degree of saturation
*n*: Porosity
*τ*: The tortuosity coefficient
*z*: (M) the depth
*r*: (M) the radius
*r*_*i*_: (Mol/kg/s) the reaction rate of component *i*
*r*_*CH*__*4*_: (Mol/kg/s) reaction rate of CH_4_
*r*_*CO*__*2*_: (Mol/kg/s) reaction rate of CO_2_
*r*_*O*__*2*_: (Mol/kg/s) reaction rate of O_2_
*ρ*_*d*_: (Kg/m^3^) the dry bulk density of the soil
*N*_*i*__*,z*_: (mol/m^2^/s) the flux of component *i* in the axial direction
*N*_*i*__*,r*_: (Mol/m^2^/s) the flux of component *i* in the radial direction
*N*_*i*__*,s*_: (Mol/m^2^/s) the total flux of component *i* in direction *s* (include *z* or *r*)
*N*_*i*__*,s*_^*D*^: (Mol/m^2^/s) diffusion flux of component *i* in direction *s* (include *z* or *r*)
*N*_*i*__*,s*_^*V*^: (Mol/m^2^/s) advection flux of component *i* in direction *s* (include *z* or *r*)
*N*_measure_: (Mol/m^2^/s) the flux calculated by the concentration–time curve
*N*_ref_: (Mol/m^2^/s) the referenced emission flux without using the chamber
*N*_Bottom_: (Mol/m^2^/s) flux at the bottom boundary
*N*_*i*__*,r*_: (Mol/m^2^/s) radial flux of component *i*.
*k*_*g*_: (M^2^) gas permeability
*k*_*gr*_: (M^2^) horizontal gas permeability
*k*_*gz*_: (M^2^) vertical gas permeability
*μ*: (Pa∙s) gas viscosity
*R*: (m^3^/Pa·K/mol) ideal gas constant
*T*: (K) absolute temperature
*D*_*ij*_: (M^2^/s) the ordinary diffusion coefficient between component *i* and *j*
*D*^*K*^_*i*_: (M^2^/s) the Knudsen diffusion coefficient for component *i*.
*N*^*D*^_*i*__*,Blanc*_: (M^2^/s) the diffusion flux for component *i* by Blanc’s model
*D*_*i*_: (M^2^/s) diffusion coefficient for the component *i* for Blanc’s model
*x*_*i*_: Molar fraction of component *i*
*x*_*i*__,__atm_: The atmospheric mole fraction of component *i*
*V*_*max*_: (Mol/kg/s) maximum reaction rate of methane oxidation
*k*_CH__4_: The half-saturation constant of CH_4_
*k*_O__2_: The half-saturation constant of and O_2_
*ε*: The relative error of flux
*p*: (Pa) total gas pressure
*p*_*atm*_: The absolute pressure
*p*_*i*_: (Mol/m^3^) the partial pressure of component *i*
*p*_*i,*__*atm*_: The absolute partial atmospheric pressure of component *i*
*p*_i,__chamber_: Partial pressure of component *i* inside the chamber

## References

[CR1] Abichou T, Mahieu K, Chanton J, Romdhane M, Mansouri I (2011). Scaling methane oxidation: from laboratory incubation experiments to landfill cover field conditions. Waste Manage.

[CR2] Ahmadi N, Heck K, Rolle M, Helmig R, Mosthaf K (2021). On multicomponent gas diffusion and coupling concepts for porous media and free flow: a benchmark study. Comput Geosci.

[CR3] Bain WG, Hutyra L, Patterson DC, Bright AV, Daube BC, Munger JW, Wofsy SC (2005). Wind-induced error in the measurement of soil respiration using closed dynamic chambers. Agric for Meteorol.

[CR4] Bear J (1972). Dynamics of fluids in porous media.

[CR5] Bian R, Komiya T, Shimaoka T, Chai X, Sun Y (2019). Simulative analysis of vegetation on CH4 emission from landfill cover soils: Combined effects of root-water uptake, root radial oxygen loss, and plant-mediated CH4 transport. J Clean Prod.

[CR6] Bian R, Shi W, Chai X, Sun Y (2020). Effects of plant radial oxygen loss on methane oxidation in landfill cover soil: A simulative study. Waste Manage.

[CR7] Bian R, Chen J, Li W, Sun Y, Chai X, Wang H, Wang Y, Zhao J (2021). Numerical modeling of methane oxidation and emission from landfill cover soil coupling water-heat-gas transfer: Effects of meteorological factors. Process Saf Environ Prot.

[CR8] Chi X, Amos RT, Stastna M, Blowes DW, Sego DC, Smith L (2013). The Diavik Waste Rock Project: implications of wind-induced gas transport. Appl Geochem.

[CR9] Christiansen JR, Korhonen JF, Juszczak R, Giebels M, Pihlatie M (2011). Assessing the effects of chamber placement, manual sampling and headspace mixing on CH 4 fluxes in a laboratory experiment. Plant Soil.

[CR10] COMSOL (2014) COMSOL Multiphysics. 5th ed. http://cn.comsol.com/release/5.0

[CR11] Cotel S, Schäfer G, Traverse S, Marzougui-Jaafar S, Gay G, Razakarisoa O (2015). Evaluation of VOC fluxes at the soil-air interface using different flux chambers and a quasi-analytical approach. Water Air Soil Pollut.

[CR12] Davidson EA, Savage KVLV, Verchot LV, Navarro R (2002). Minimizing artifacts and biases in chamber-based measurements of soil respiration. Agric for Meteorol.

[CR13] De Visscher A, Van Cleemput O (2003). Simulation model for gas diffusion and methane oxidation in landfill cover soils. Waste Manage.

[CR14] Detto M, Verfaillie J, Anderson F, Xu L, Baldocchi D (2011). Comparing laser-based open-and closed-path gas analyzers to measure methane fluxes using the eddy covariance method. Agric for Meteorol.

[CR15] Ding L, Lu Q, Wang C, Shi Z, Cao W, Li B (2015). Effects of configuration and headspace mixing on the accuracy of closed chambers for dairy farm gas emission measurement. Appl Eng Agric.

[CR16] Dlugokencky EJ, Bruhwiler L, White JWC, Emmons LK, Novelli PC, Montzka SA, Masarie KA, Lang PM, Crotwell AM, Miller JB, Gatti LV (2009). Observational constraints on recent increases in the atmospheric CH4 burden. Geophys Res Lett.

[CR17] Duan Z, Kjeldsen P, Scheutz C (2021). Trace gas composition in landfill gas at Danish landfills receiving low-organic waste. Waste Manage.

[CR18] Fallah B, Torabi F (2021). Application of periodic parameters and their effects on the ANN landfill gas modeling. Environ Sci Pollut Res.

[CR19] Farkas C, Alberti G, Balogh J, Barcza Z, Birkás M, Czóbel S, Davis KJ, Fuehrer E, Gelybo G, Grosz B, Kljun N, Koos S, Machon A, Marjanovic H, Nagy Z, Peressotti A, Pinter K, Toth E, Horvath L (2011). Methodologies. Atmospheric Greenhouse Gases: The Hungarian Perspective.

[CR20] Fen CS (2006). Effective gas-phase diffusion coefficient in soils. WIT Transactions on Ecology and the Environment, 94.

[CR21] Fen CS (2014). Assessing Vadose Zone Biodegradation by a Multicomponent Gas Transport Model. Vadose Zone J.

[CR22] Feng S, Ng CWW, Leung AK, Liu HW (2017). Numerical modelling of methane oxidation efficiency and coupled water-gas-heat reactive transfer in a sloping landfill cover. Waste Manage.

[CR23] Feng S, Leung AK, Liu HW, Ng CWW, Zhan LT, Chen R (2019). Effects of thermal boundary condition on methane oxidation in landfill cover soil at different ambient temperatures. Sci Total Environ.

[CR24] Feng Y, Mousavi MS, Eun J (2020). Field Monitoring of Landfill Gas Emissions through an Intermediate Cover with Co-Extruded EVOH Geomembrane in an Operating Landfill. Geo-Congress 2020: Geo-Systems, Sustainability, Geoenvironmental Engineering, and Unsaturated Soil Mechanics.

[CR25] Feng SJ, Zhu ZW, Chen ZL, Chen HX (2020). Analytical model for multicomponent landfill gas migration through four-layer landfill biocover with capillary barrier. Int J Geomech.

[CR26] Fujikawa T, Miyazaki T (2005). Effects of bulk density and soil type on the gas diffusion coefficient in repacked and undisturbed soils. Soil Sci.

[CR27] Garg A, Bordoloi S, Ni J, Cai W, Maddibiona PG, Mei G, Poulsen TG, Lin P (2019). Influence of biochar addition on gas permeability in unsaturated soil. Géotechnique Letters.

[CR28] Gebert J, Rachor I, Gröngröft A, Pfeiffer EM (2011). Temporal variability of soil gas composition in landfill covers. Waste Manage.

[CR29] Gonzalez-Valencia R, Magana-Rodriguez F, Maldonado E, Salinas J, Thalasso F (2015). Detection of hotspots and rapid determination of methane emissions from landfills via a ground-surface method. Environ Monit Assess.

[CR30] Gutiérrez MC, Siles JA, Diz J, Chica AF, Martín MA (2017). Modelling of composting process of different organic waste at pilot scale: Biodegradability and odor emissions. Waste Manage.

[CR31] Haro K, Ouarma I, Nana B, Bere A, Tubreoumya GC, Kam SZ, Lavilleb P, Loubet B, Koulidiati J (2019). Assessment of CH4 and CO2 surface emissions from Polesgo's landfill (Ouagadougou, Burkina Faso) based on static chamber method. Adv Clim Chang Res.

[CR32] He PJ, Tang JF, Yang N, Fang JJ, He X, Shao LM (2012). The emission patterns of volatile organic compounds during aerobic biotreatment of municipal solid waste using continuous and intermittent aeration. J Air Waste Manag Assoc.

[CR33] Hibi Y, Fujinawa K, Nishizaki S, Okamura K, Tasaki M (2009). Multi-component migration in the gas phase of soil: comparison between results of experiments and simulation by dusty gas model. Soils Found.

[CR34] Ho CK, Webb SW (2006). Gas transport in porous media (Vol. 20).

[CR35] Huang D, Du Y, Xu Q, Ko JH (2022). Quantification and control of gaseous emissions from solid waste landfill surfaces. J Environ Manage.

[CR36] Huang H, Cai WL, Zheng Q, Chen PN, Huang CR, Zeng QJ, Himanshu K, Zhu HH, Ankit G and Kushvaha V. (2020). Gas permeability in soil amended with biochar at different compaction states. In IOP Conference Series: Earth and Environmental Science. 463(1):012073. IOP Publishing.

[CR37] Izumoto S, Hamamoto S, Kawamoto K, Nagamori M, Nishimura T (2018). Monitoring of methane emission from a landfill site in daily and hourly time scales using an automated gas sampling system. Environ Sci Pollut Res.

[CR38] Janssens IA, Kowalski AS, Longdoz B, Ceulemans R (2000). Assessing forest soil CO2 efflux: an in situ comparison of four techniques. Tree Physiol.

[CR39] Jeong S, Park J, Kim YM, Park MH, Kim JY (2019). Innovation of flux chamber network design for surface methane emission from landfills using spatial interpolation models. Sci Total Environ.

[CR40] Krishna R, Wesselingh JA (1997). The Maxwell-Stefan approach to mass transfer. Chem Eng Sci.

[CR41] Laemmel T, Mohr M, Schack-Kirchner H, Schindler D, Maier M (2017). Direct observation of wind-induced pressure-pumping on gas transport in soil. Soil Sci Soc Am J.

[CR42] Li GY, Dai S, Zhan LT, Chen YM (2019). A pore-scale numerical investigation of the effect of pore characteristics on flow properties in soils. J Zhejiang Univ-Sci A.

[CR43] Li H, Meng B, Yue B, Gao Q, Ma Z, Zhang W, Li T, Yu L (2020). Seasonal CH4 and CO2 effluxes in a final covered landfill site in Beijing. China Sci Total Environ.

[CR44] Liu Y, Wang C, Ding L, Wang Z, Teng G, Shi Z, Li B (2019). Influence of deployment time and surface wind speed on the accuracy of measurements of greenhouse gas fluxes using a closed chamber method under low surface wind speed. J Air Waste Manag Assoc.

[CR45] Livingston GP, Hutchinson GL, Spartalian K (2005). Diffusion theory improves chamber-based measurements of trace gas emissions. Geophys Res Lett.

[CR46] Maier M, Schack-Kirchner H (2014). Using the gradient method to determine soil gas flux: A review. Agric for Meteorol.

[CR47] Mason EA, Malinauskas AP (1983). Gas transport in porous media: the dusty-gas model.

[CR48] Moldrup P, Olesen T, Gamst J, Schjønning P, Yamaguchi T, Rolston DE (2000). Predicting the gas diffusion coefficient in repacked soil water-induced linear reduction model. Soil Sci Soc Am J.

[CR49] Ng CWW, Feng S, Liu HW (2015). A fully coupled model for water–gas–heat reactive transport with methane oxidation in landfill covers. Sci Total Environ.

[CR50] Ngusale GK, Oloko MO, Otiende F, Aguko KP (2021). Estimation of methane and landfill gas emission from an open dump site. Int J Environ Waste Manage.

[CR51] Pape L, Ammann C, Nyfeler-Brunner A, Spirig C, Hens K, Meixner FX (2009). An automated dynamic chamber system for surface exchange measurement of non-reactive and reactive trace gases of grassland ecosystems. Biogeosciences.

[CR52] Parkin TB, Venterea RT, Hargreaves SK (2012). Calculating the detection limits of chamber-based soil greenhouse gas flux measurements. J Environ Qual.

[CR53] Perera MD, Hettiaratchi JP, Achari G (2002). A mathematical modeling approach to improve the point estimation of landfill gas surface emissions using the flux chamber technique. J Environ Eng Sci.

[CR54] Pierini VI, Bartoloni N, Ratto SE (2018). Greenhouse gases emissions from a closed old landfill cultivated with biomass crops. Environ Dev Sustain.

[CR55] Pihlatie MK, Christiansen JR, Aaltonen H, Korhonen JFJ, Nordbo A, Rasilo T, Benanti G, Giebels M, Helmy M, Sheehy J, Jones S, Juszczak R, Klefoth R, Lobo-do-Vale R, Rosa AP, Schreiber P, Serça D, Vicca S, Wolf B, Pumpanen J (2013). Comparison of static chambers to measure CH4 emissions from soils. Agric for Meteorol.

[CR56] Pinheiro LT, Cattanio JH, Imbiriba B, Castellon SEM, Elesbão SA, Ramos JRS (2019). Carbon Dioxide and methane flux measurements at a large unsanitary dumping site in the Amazon Region. Rev Bras De Ciênc Ambientais.

[CR57] Prajapati P, Santos EA (2018). Estimating methane emissions from beef cattle in a feedlot using the eddy covariance technique and footprint analysis. Agric for Meteorol.

[CR58] Redeker KR, Baird AJ, Teh YA (2015). Quantifying wind and pressure effects on trace gas fluxes across the soil–atmosphere interface. Biogeosciences.

[CR59] Reid RC, Prausnitz JM, Poling BE (1987). The properties of gases and liquids.

[CR60] Rochette P, Hutchinson GL (2005). Measurement of soil respiration in situ: chamber techniques. Micrometeorology Agricultural Systems.

[CR61] Sahoo BK, Mayya YS (2010). Two dimensional diffusion theory of trace gas emission into soil chambers for flux measurements. Agric for Meteorol.

[CR62] Senevirathna DG, Achari G, Hettiaratchi JP (2006). A laboratory evaluation of errors associated with the determination of landfill gas emissions. Can J Civ Eng.

[CR63] Senevirathna DGM, Achari G, Hettiaratchi JPA (2007). A mathematical model to estimate errors associated with closed flux chambers. Environ Model Assess.

[CR64] Shen S, Chen Y, Zhan L, Xie H, Bouazza A, He F, Zuo X (2018). Methane hotspot localization and visualization at a large-scale Xi'an landfill in China: Effective tool for landfill gas management. J Environ Manage.

[CR65] Takle ES, Massman WJ, Brandle JR, Schmidt RA, Zhou X, Litvina IV, Garcia R, Doyle G, Rice CW (2004). Influence of high-frequency ambient pressure pumping on carbon dioxide efflux from soil. Agric for Meteorol.

[CR66] Tamminen J, Ahonen T, Ahola J, Hammo S (2016). Fan pressure-based testing, adjusting, and balancing of a ventilation system. Energ Effi.

[CR67] Thorstenson DC, Pollock DW (1989). Gas transport in unsaturated zones: multicomponent systems and the adequacy of Fick’s laws. Water Resour Res.

[CR68] US EPA (US Environmental Protection Agency) (2004) Measurement of Gaseous Emission rated from Land Surfaces using an Emission Isolation Flux Chamber. User’s guide. EPA 600/8-86-008 (NTIS PB-223161). US EPA, Washington

[CR69] Venterea RT (2013). Theoretical comparison of advanced methods for calculating nitrous oxide fluxes using non-steady state chambers. Soil Sci Soc Am J.

[CR70] Venterea RT, Baker JM (2008). Effects of soil physical nonuniformity on chamber-based gas flux estimates. Soil Sci Soc Am J.

[CR71] Venterea RT, Spokas KA, Baker JM (2009). Accuracy and precision analysis of chamber-based nitrous oxide gas flux estimates. Soil Sci Soc Am J.

[CR72] Venterea RT, Petersen SO, De Klein CA, Pedersen AR, Noble AD, Rees RM, Gamble JD, Parkin TB (2020). Global Research Alliance N2O chamber methodology guidelines: Flux calculations. J Environ Qual.

[CR73] Wang X, Jia M, Lin X, Xu Y, Ye X, Kao CM, Chen S (2017). A comparison of CH4, N2O and CO2 emissions from three different cover types in a municipal solid waste landfill. J Air Waste Manag Assoc.

[CR74] Wang Q, Zuo X, Xia M, Xie H, He F, Shen S, Bouazza A, Zhu L (2019). Field investigation of temporal variation of volatile organic compounds at a landfill in Hangzhou. China Environ Sci Pollut Res.

[CR75] Wang Q, Fei S, Wang L, Bouazza A, Shen S, Xie H (2020). Investigation of methane fluxes from temporary cover of Xi’an Jiangcungou landfill, China. Environ Geotech.

[CR76] Webb SW, Pruess K (2003). The use of Fick's law for modeling trace gas diffusion in porous media. Transp Porous Media.

[CR77] Wickramarachchi P, Kawamoto K, Hamamoto S, Nagamori M, Moldrup P, Komatsu T (2011). Effects of dry bulk density and particle size fraction on gas transport parameters in variably saturated landfill cover soil. Waste Manage.

[CR78] Winton RS, Richardson CJ (2016). A cost-effective method for reducing soil disturbance-induced errors in static chamber measurement of wetland methane emissions. Wetlands Ecol Manage.

[CR79] Xu XH, Yang YP, Wang DH (2003). CH4 emission and recovery from Municipal Solid Waste in China. J Zhejiang Univ-SCI A.

[CR80] Xu L, Furtaw MD, Madsen RA, Garcia RL, Anderson DJ, McDermitt DK (2006). On maintaining pressure equilibrium between a soil CO2 flux chamber and the ambient air. J Geophys Res: Atmos.

[CR81] Yilmaz M, Tinjum JM, Acker C, Marten B (2021). Transport mechanisms and emission of landfill gas through various cover soil configurations in an MSW landfill using a static flux chamber technique. J Environ Manage.

[CR82] Zhan LT, Qiu QW, Xu WJ, Chen YM (2016). Field measurement of gas permeability of compacted loess used as an earthen final cover for a municipal solid waste landfill. J Zhejiang Univ-SCI A.

[CR83] Zhan LT, Wu T, Feng S, Lan JW, Chen YM (2020). A simple and rapid in situ method for measuring landfill gas emissions and methane oxidation rates in landfill covers. Waste Manage Res.

[CR84] Zhang C, Guo Y, Wang X, Chen S (2019). Temporal and spatial variation of greenhouse gas emissions from a limited-controlled landfill site. Environ Int.

[CR85] Zhao J, Zhang M, Xiao W, Wang W, Zhang Z, Yu Z, Xiao Q, Cao Z, Xu J, Zhang X, Liu S (2019). An evaluation of the flux-gradient and the eddy covariance method to measure CH4, CO2, and H2O fluxes from small ponds. Agric for Meteorol.

[CR86] Zuo X, Chen Y, Wang L, Xie H, Shen S (2020). Multicomponent landfill gas transport in soil cover: column tests and numerical modelling. Environ Geotech.

